# Cerebellar transcranial current stimulation – An intraindividual comparison of different techniques

**DOI:** 10.3389/fnins.2022.987472

**Published:** 2022-09-15

**Authors:** Rebecca Herzog, Till M. Berger, Martje G. Pauly, Honghu Xue, Elmar Rueckert, Alexander Münchau, Tobias Bäumer, Anne Weissbach

**Affiliations:** ^1^Institute of Systems Motor Science, University of Lübeck, Lübeck, Germany; ^2^Department of Neurology, University Hospital Schleswig Holstein, Lübeck, Germany; ^3^Institute of Neurogenetics, University of Lübeck, Lübeck, Germany; ^4^Institute for Robotics and Cognitive Systems, University of Lübeck, Lübeck, Germany; ^5^Montanuniversität Leoben, Leoben, Austria

**Keywords:** cerebellum, plasticity, tRNS, tACS, tDCS, TMS, IMU

## Abstract

Transcranial current stimulation (tCS) techniques have been shown to induce cortical plasticity. As an important relay in the motor system, the cerebellum is an interesting target for plasticity induction using tCS, aiming to modulate its excitability and connectivity. However, until now it remains unclear, which is the most effective tCS method for inducing plasticity in the cerebellum. Thus, in this study, the effects of anodal transcranial direct current stimulation (tDCS), 50 Hz transcranial alternating current stimulation (50 Hz tACS), and high frequency transcranial random noise stimulation (tRNS) were compared with sham stimulation in 20 healthy subjects in a within-subject design. tCS was applied targeting the cerebellar lobe VIIIA using neuronavigation. We measured corticospinal excitability, short-interval intracortical inhibition (SICI), short-latency afferent inhibition (SAI), and cerebellar brain inhibition (CBI) and performed a sensor-based movement analysis at baseline and three times after the intervention (post1 = 15 min; post2 = 55 min; post3 = 95 min). Corticospinal excitability increased following cerebellar tACS and tRNS compared to sham stimulation. This effect was most pronounced directly after stimulation but lasted for at least 55 min after tACS. Cortico-cortical and cerebello-cortical conditioning protocols, as well as sensor-based movement analyses, did not change. Our findings suggest that cerebellar 50 Hz tACS is the most effective protocol to change corticospinal excitability.

## Introduction

The cerebellum plays an important role in sensorimotor integration, cognitive tasks, and emotional processing (Stoodley and Schmahmann, 2009), rendering it an interesting target region for non-invasive brain stimulation (NIBS) to alter its excitability and connectivity. Beside repetitive transcranial magnetic stimulation, transcranial current stimulation (tCS) paradigms are NIBS techniques, which are based on the application of a low-intensity current flow between two electrodes. The tCS technique currently most commonly used is transcranial direct current stimulation (tDCS). However, the effects of tDCS show large inter- and intra-individual variability (Batsikadze et al., 2019). Therefore, other methods including transcranial alternating current stimulation (tACS) and transcranial random noise stimulation (tRNS) might be interesting alternatives (Naro et al., 2017; Miyaguchi et al., 2020).

TACS has already been used for cerebellar stimulation with frequencies ranging from 5 Hz to 300 Hz with different results (Naro et al., 2016, 2017; Miyaguchi et al., 2019, 2020; Wessel et al., 2020; Schubert et al., 2021; Spampinato et al., 2021). One reason for using different frequencies is related to the fact that different target cell populations have different oscillatory properties. It has been shown that when using low intensities, stimulation frequencies close to the endogenous oscillation frequency induce the strongest spiking resonance at a cellular level (Reato et al., 2010). When using higher intensities, effects can also be induced at subharmonics, and the power of the endogenous frequency may be modulated (Reato et al., 2010, 2013). As Purkinje cells have an endogenous firing rate of about 50 Hz (Raman and Bean, 1999) and play an important role in coordination, 50 Hz seems to be a promising frequency to influence movements and coordination, especially when using low intensities. However, concerning 50 Hz, previous studies revealed different results. Whereas motor performance of the upper limb was reported to be improved after offline tACS (Naro et al., 2017), online tACS it did not improve or even impaired the acquisition of a motor skill when used online (Wessel et al., 2020; Giustiniani et al., 2021). Also, neurophysiological results differed between studies: While Naro et al. (2016) found a reduction of cerebellar brain inhibition (CBI) and an increase of motor evoked potential (MEP) amplitudes, other studies showed no changes in cortical excitability (Spampinato et al., 2021). Reasons for these differences are probably related to the use of different protocols including on-/offline stimulation, which is very relevant because of the known state-dependency of tACS effects (Feurra et al., 2013), stimulation duration, and different methods to investigate CBI.

In contrast to tACS, during tRNS the current alternates with randomized frequencies ranging from 0.1 to 640 Hz. In a recent study, 20 min of left primary motor cortex (M1) tRNS stimulation was more efficient in increasing motor cortex excitability compared to tDCS and 140 Hz tACS (Inukai et al., 2016), which was probably mediated by the high-frequency spectrum (> 100 Hz) (Terney et al., 2008). Due to its high efficacy when applied to M1, positive findings following the application to other brain regions including the dorsolateral prefrontal cortex (Pena et al., 2019) and its possible differential effect regarding location and frequency of the stimulation (Campana et al., 2016), tRNS might be a promising tool for cerebellar stimulation. Given the limited experience with tRNS and its mode of action, the expected effects are currently largely unclear, so its use has to be considered exploratory.

To our knowledge, due to the high variability of study protocols and modes of application used in different studies no standardized comparison has been carried out between tDCS, tACS, and tRNS in the same study population (Kumari et al., 2019; Antal et al., 2022). To fill this gap, we directly compared anodal tDCS, 50 Hz tACS, and high-frequency tRNS (hf-tRNS) with sham stimulation as to their efficacy to induce plasticity in the cerebellum.

We examined the size of MEPs because MEPs reflect corticospinal excitability, which has been shown to be increased following tACS (Naro et al., 2017). To investigate the excitability of the cerebello-thalamo-cortical pathway, we included CBI as an additional readout. To test CBI, a conditioning stimulus is applied to the cerebellum before stimulating the contralateral M1, which decreases cortical excitability reflected in reduced MEP amplitudes (Ugawa et al., 1995; Werhahn et al., 1996). This effect is thought to be mediated by the activation of Purkinje cells reducing the excitatory drive of dentato-thalamo-cortical pathways (Celnik, 2015). CBI can thus be taken as a marker of Purkinje cell excitability, which is likely modulated by tCS, e.g., following 50 Hz tACS (Naro et al., 2017). We also tested short-interval intracortical inhibition (SICI), where a subthreshold conditioning stimulus over M1 precedes a suprathreshold pulse given to M1 by 1 to 6 ms (Berardelli et al., 2008). It is a measure of GABA A-ergic mediated M1 inhibition and has been documented to be modifiable by cerebellar tDCS and a conditioning cerebellar TMS pulse, but not by 50 Hz tACS (Daskalakis et al., 2004; Ates et al., 2018; Spampinato et al., 2021). The effects of tRNS on SICI are currently unclear. Because of the important role of the cerebellum for sensorimotor processing (Dubbioso et al., 2015; Yildiz et al., 2018) we also included short-latency afferent inhibition (SAI) where a peripheral nerve is stimulated before stimulation of the contralateral M1 (Chen et al., 1999) as a readout. Finally, to capture tCS effects on motor performance, we measured arm movements using inertial measurement units (IMUs). In previous studies, kinematic measurements including IMUs or optoelectronic devices have successfully been used to investigate patients with cerebellar dysfunction including those with ataxia and dystonia (Bologna et al., 2016; Krishna et al., 2019). Thus, to capture the effects of the different tCS techniques on the excitability/activity of the cerebellum and interconnected pathways we used a comprehensive battery of behavioral and neurophysiological measures.

## Materials and methods

### Study design, participants, and questionnaire

We investigated 20 healthy, right-handed subjects (12 female, mean age 23 years; range: 20–31 years) without self-reported neurologic or psychiatric disorders. All of them were examined at four different time points to evaluate the effects of three different cerebellar tCS techniques (i.e., anodal tDCS, 50 Hz tACS, and high-frequency tRNS) in comparison to sham stimulation. The interval between interventions was at least 1 week to avoid carry-over effects. Measurements were performed at the same time of the day (either at 9 am or 1 pm) in every session of each subject. The order of interventions was randomized between subjects. The subjects were blinded regarding the used tCS method.

**FIGURE 1:**
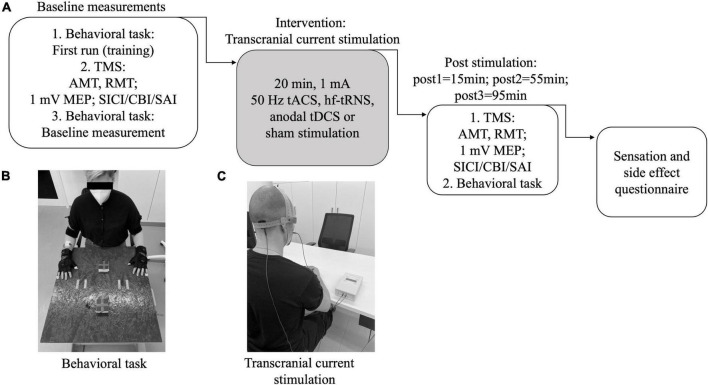
Study design. **(A)** Experimental setup: In every session the timetable was as shown here. Before and after the plasticity induction the same measurements were performed. After the whole day, a questionnaire was answered. AMT, active motor threshold; RMT, resting motor threshold; MEP, motor evoked potential; SICI, short-interval intracortical inhibition; SAI, short-latency afferent inhibition; CBI, cerebellar brain inhibition; tACS, transcranial alternating current stimulation; tDCS, transcranial direct current stimulation; (hf-)tRNS, (high-frequency) transcranial random noise stimulation. **(B)** Experimental setup for behavioral task. **(C)** Setup for transcranial current stimulation.

Every session began with a training of the motor task, as described below. Before and three times after tCS (pre = before stimulation; post1 = 15 min; post2 = 55 min; post3 = 95 min) (see Figure 1A) we determined resting and active motor thresholds (RMT and AMT) and MEP amplitudes. We also performed dual-pulse paradigms to evaluate SICI, SAI, and CBI. In addition, three-dimensional (X, Y, Z) acceleration profiles of wrists and hands during a motor task were analyzed using sensors. Measurements were performed in the same order at all time points as follows: (i) MEP, RMT, and AMT; (ii) paired pulse measurements; (iii) movement task. All conditioned MEPs were collected in randomized order within one measurement. Cerebellar stimulation was performed on the right side, e.g., contralateral to the left M1 stimulation. The movement analyses were performed bilaterally.

After each tCS session, all participants completed a questionnaire addressing the side effects of cerebellar stimulation in general (vertigo, change in coordination ability), of tCS (local heat, skin sensations), and of TMS (headache). All side effects had to be evaluated on a visual analog scale from 0 to 10 with 0 indicating “no effect” and 10 “the strongest imaginable effect.”

Additionally, the participants were asked on their last day, which day they believed to be the day of sham stimulation, and which measurement day was the most/the least comfortable. The questionnaire is available as Supplementary material.

### Neuronavigation

To precisely target the stimulation site previously identified in an individual T1-weighted MRI scan, we used the Brainsight neuronavigation system (Rogue Research, Montreal, Canada) in combination with the Polaris camera (Northern Digital, Ontario, Canada). The stimulation site for M1 was marked at the hand knob, but was always verified by determining the neurophysiological “motor hot spot,” i.e., the location where TMS pulses administered at a suprathreshold intensity consistently produced the largest MEPs.

For cerebellar stimulation, we chose lobule VIIIA, because it has been shown to be important for the execution of motor tasks and learning processes (Stoodley and Schmahmann, 2009; Guell and Schmahmann, 2020). Due to its superficial location, it is reachable *via* TMS and tCS and has already been used as a target in other TMS studies (Popa et al., 2013).

### Transcranial magnetic stimulation measurements

#### Experimental setup

The experimental setup for TMS was similar to previous published TMS studies (Weissbach et al., 2015, 2017). Each subject was seated in a comfortable position with the arms positioned on a pillow if necessary to avoid muscle tension. Additionally, participants were regularly instructed to relax their body with open eyes. Electromyography (EMG) was measured over the right first dorsal interosseus muscles (FDI) by Ag/Ag-Cl disk surface electrodes in a belly tendon montage. The earth electrode was fixed at the wrist. A D360 amplifier (Digitimer Limited, Welwyn Garden City, Hertfordshire, United Kingdom) was used to filter (20 Hz and 2 kHz) and amplify EMG signals. With a laboratory interface (Micro 1401; Cambridge Electronics Design (CED), Cambridge, United Kingdom) the EMG signal, which was sampled at 5 kHz, was digitized and recorded. Data was stored on a personal computer using the SIGNAL software (Cambridge Electronic Devices, Cambridge, United Kingdom).

#### Single-pulse and conditioned transcranial magnetic stimulation

TMS pulses were generated by two Magstim 200^2^ and one Magstim 200 magnetic stimulator (Magstim Company, Whitland, Dyfed, United Kingdom). Left M1 and right cerebellum were stimulated by a 70 mm figure-of-eight-shaped coil (Magstim Company, Whitland, Dyfed, United Kingdom). For cerebellar stimulation, the coil was positioned tangentially to the scalp with the handle directed upward, as it has frequently been used in previous studies to examine CBI (Carrillo et al., 2013; Brusa et al., 2014; Koch et al., 2014; Benussi et al., 2018, 2019). We did not opt for a double-cone coil, since our participants did not tolerate such stimulation due to pain and discomfort.

MEPs were generated by a suprathreshold intensity evoking an MEP of about 1 mV. Before and after the intervention, the same stimulator output was used for the measurements of unconditioned MEPs. RMT was defined as the lowest intensity capable to produce 5 out of 10 MEPs with an amplitude between 50 and 100 μV at a resting FDI. AMT was defined as the lowest possible intensity required to produce 5 out of 10 MEPs > 150 μV at an activated FDI with 10% of maximum voluntary contraction using a Martin-Balloon-Vigorimeter (KLS Martin, Tuttlingen, Germany). Cerebello-M1 interaction was probed with an interstimulus interval (ISI) of 5 ms and an intensity of the CP of 90% RMT (Carrillo et al., 2013; Brusa et al., 2014; Koch et al., 2014; Benussi et al., 2018, 2019). For the unconditioned MEPs, as reference for the conditioned MEPs, the test stimulus (TS) intensity was adjusted to produce an amplitude of 1 mV before and after plasticity induction.

For SAI, TMS pulses were preceded by conditioning electrical pulses of the right index finger with an ISI of 25 ms. A pair of ring electrodes was placed with the cathode at the proximal part of the right index finger and the anode 2 cm distally at the middle part of the finger. Electrical stimulation was performed using a Digitimer Constant Current DS7A Stimulator (Digitimer Limited, United Kingdom) and consisted of a brief pulse (0.1 ms duration, 500 V) with an intensity of threefold the individual’s sensory perception threshold. The threshold was defined as the lowest current intensity that was regularly detected by the subject (Tokimura et al., 2000; Ganos et al., 2014; Weissbach et al., 2015, 2022; Turco et al., 2021). The target MEP amplitude was 1 mV. For SICI measurements, a conditioning stimulus (CS) (100% AMT) was applied with an ISI of 3 ms before a suprathreshold TS that was set to elicit an MEP response of about 1 mV as described before (Brown et al., 2019). Given that the AMT is lower than the RMT and SICI was tested at rest, CS with 100% AMT did not induce MEP, so we ensured that the CS was subthreshold (as required by the definition of SICI) (Berardelli et al., 2008).

### Sensor-based movement analysis

For movement analysis, all participants performed an easy task, where they had to alternately touch two fixed points, which were 30 cm apart from each other. We opted for this task because we wanted to avoid additional learning/plasticity effects. Additionally, for sensor-based analysis it is important to limit the degrees of freedom. The task was adapted from the finger-nose test of the Scale for the Assessment and Rating of Ataxia (SARA), which is frequently used to measure clinical cerebellar symptoms in ataxia patients. To capture the participants motion trajectories, IMUs (aktos-t sensor, myon, Schwarzenberg, Switzerland), were fixed on both hands and wrists (see Figure 1B). The participants were instructed to perform repetitive tapping with both hands (one after the other) between two fixed sensor-pads (which provided a binary signal indicating a physical contact; distance: 30 cm) in two different directions (horizontal/vertical) and two different velocities (representing different difficulty levels; 1,5 Hz; as fast as possible). In every task, 30 tapping cycles were performed. Every task was recorded separately. We recorded eight different tasks at each time point. For further analysis, we used Probabilistic Movement Primitives (ProMP) (Paraschos et al., 2013), which are widely used in modeling robot motions (Paraschos et al., 2017; Gomez-Gonzalez et al., 2016) and human motions (Lim et al., 2005; Kohlschuetter et al., 2016). In contrast to conventional approaches (Bologna et al., 2016; Kwak et al., 2020; Markovic et al., 2020), ProMPs, as a Machine Learning approach, can effectively learn the features describing the trajectory shape automatically. Compared to other deterministic models providing only the mean values, a probabilistic characterization is more informative and robust. The general principle of ProMPs is as follows: It assumes a weighted combination of a set of pre-specified basis functions distributed in time (i.e., features). The weights of each feature can be directly learned from the demonstrations and be fitted to trajectories featuring arbitrary shapes. To demonstrate the level of difference between two sets of trajectories, which corresponds to the comparison sets we would investigate, further probabilistic distance measures can be applied to the learned feature space. Before using ProMPs to analyze the trajectories, data were post-processed using segmentation, alignment, and normalization over time. With the learned weights of ProMPs as a feature representation, we performed a symmetric version of Kullback-Leibler divergence (KL-divergence) (Johnson and Sinanovic, 2001) between sets of trajectories to measure the effect of plasticity induction [for details see Xue et al. (2021)]. The KL-divergence describes the difference between two probability distributions. Additionally, we used the standard deviation to measure the exactness of movements. All in all, the ProMPs are used to find distinct differences in the three-dimensional movement patterns, although the features are not directly convertible in specific movements themselves, but an abstract description of movement properties.

### Transcranial current stimulation/plasticity induction

Anodal tDCS, 50 Hz tACS, and high frequency tRNS (with frequencies between 100 and 640 Hz) were used and compared to sham stimulation.

For all tCS methods, we used the same electrode montage: Using the Brainsight neuronavigation system the anode was placed to target the right cerebellar lobule VIIIA, the cathode was placed on the right cheek (masseter muscle) (see Figure 1C). This montage [“Celnik-Montage”; (Galea et al., 2009)] was frequently used in the past (Wessel et al., 2016; Jackson et al., 2019; Spampinato et al., 2021) and has been shown to generate an efficient and side-specific electric field (Rezaee and Dutta, 2019).

Stimulation was performed with a DC-Stimulator plus (neuroCare, Munich, Germany) for 20 min with an intensity of 1 mA, transmitted over 3 cm × 3 cm rubber electrodes evenly covered with conductive paste (Ten20 conductive electrode paste; Weaver and Company, United States), so current density was 0.11 mA/cm^2^.

For tDCS and tRNS, fade in/out was set to 10 s, for 50 Hz tACS to 100 cycles (2 s) because it was technically not possible to set it on 500 cycles. Setting down fading in/out on 2 s for tDCS/tRNS was not possible due to painful sensations. Apart from that, all stimulation parameters were the same to generate comparable stimulation conditions.

For sham stimulation, we used tDCS, which faded out after 60 s of stimulation, a method that has previously been used (Gandiga et al., 2006; Jackson et al., 2019).

During the stimulation, the participants remained seated, with eyes open, having the possibility to eat or drink something, and were allowed to ask questions.

### Data analysis and statistical analysis

MEP peak-to-peak amplitudes were measured in each trial. Conditioned/dual-pulse MEPs were expressed as a percentage of unconditioned MEPs. For statistical analysis, multifactorial analysis of variance with repeated measures (ANOVA) using the factors INTERVENTION (sham, tACS, tRNS, tDCS) and TIME (pre, post1, post2, post3) was performed. If ANOVA resulted in a significant *p*-value (*p* ≤ 0.05) for INTERVENTION, follow-up ANOVA was performed separately for each method with the factor TIME performing Bonferroni-Holm-corrected student *t*-test for *post hoc* testing. For dual-pulses, an additional ANOVA using the factors INTERVENTION (sham, tACS, tRNS, tDCS), TIME (pre, post1), and CONDITIONING (conditioned, unconditioned) was performed to analyze the effect of the CS.

For sensor data, we calculated the standard deviation of all averaged trajectories as a marker for exactness and performed an ANOVA with those data using the following factors: INTERVENTION (sham, tACS, tRNS, tDCS), TIME (pre, post1, post2, post3), DIRECTION (horizontal, vertical), VELOCITY (fast, slow), and HAND (right, left). To better distinguish the effects, we also calculated separated ANOVAs for right- and left-hand experiments as well as fast and slow movements with the same factors except for HAND or VELOCITY, respectively. To test for potential effects over time between interventional blocks we performed a multifactorial ANOVA comparing pre-interventional data using the factors DAY (Day 1–4), VELOCITY (fast/slow), HAND (right/left), and DIRECTION (vertical/horizontal). For further analysis, we fit the sensor data using ProMPs and characterized the difference between two sets of trajectories using symmetric KL-divergence for each participant, each post-stimulation phase, each stimulation approach, and each experimental configuration, respectively. By that, we detected corrupted data (datasets where technical problems occurred) and outliers defined as cases, where symmetric KL-divergence value exceeded the confidence level of 3σ rules. Due to the unfavorable distribution of outliers (4%, but in 30% of the subjects) we did not perform ANOVA with KL-divergences but narrowed descriptive statistics using mean values and standard error.

To analyze the recognition of sham as well as the most/least comfortable stimulation we performed X^2^ Goodness of fit test with 5 variables (sham, tACS, tRNS, tDCS, n.a.), hypothesizing a balanced frequency.

### Ethical statement

The experimental procedure was approved by the local Ethics Committee of the University of Lübeck and performed according to the ethical standards laid down in the Declaration of Helsinki. Informed and written consent was obtained from all participants.

## Results

### Effects on unconditioned motor evoked potentials and motor thresholds

Raw data for RMT, AMT, and TS (MEP) are given in Supplementary Table 1.

**FIGURE 2:**
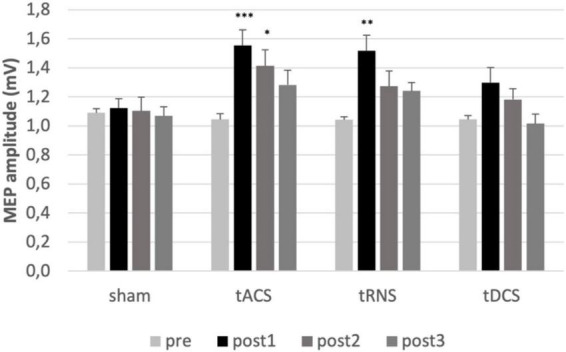
Analysis of MEPs. MEPs mean values pre and post interventions are shown. Error bars representing the standard error of the mean. *Represents a *p*-value < 0.05, ^**^ represents *p*-value < 0.01 and ^***^ *p*-value < 0.001 in Bonferroni-Holm-corrected *t*-test. Significant increase of MEP after tACS at post1 compared to pre (*p* = 0.001) and post2 compared to pre (*p* = 0.018). Significant increase of MEP after tRNS at post1 compared to pre (*p* = 0.002). MEP, motor evoked potential; FDI, first dorsal interosseous muscles; tACS, transcranial alternating current stimulation; tDCS, transcranial direct current stimulation; tRNS, transcranial random noise stimulation. post1 = 15 min; post2 = 55 min; post3 = 95 min post intervention.

Multifactorial ANOVA of MEP amplitudes showed a main effect of TIME [F(2.36) = 19.56, *p* ≤ 0.001, η^2^_p_ = 0.507], INTERVENTION [F(3) = 3.60, *p* = 0.019, η^2^_p_ = 0.159], and an interaction of INTERVENTION and TIME [F(9) = 2.75, *p* = 0.005, η^2^_p_ = 0.126] indicating that the interventions have different effects on corticospinal excitability. Analyzing the interventions separately, ANOVAs revealed time effects for tACS [F(3) = 8.65, *p* < 0.001, η^2^_p_ = 0.313], tRNS [F(3) = 8.68, *p* < 0.001, η^2^_p_ = 0.314] and tDCS [F(2.01) = 6.5, *p* = 0.004, η^2^_p_ = 0.255], but not for sham [F(3) = 0.198, *p* = 0.897, η^2^_p_ = 0.010]. *Post hoc* test revealed an increase of unconditioned MEP amplitudes after tACS (*p* = 0.001) and tRNS (*p* = 0.002) compared to the baseline measurements at post1. tACS effects persisted up to 55 min after stimulation (post2 compared to pre; *p* = 0.022). Sham stimulation had no effect. There was no significant effect comparing post1/2/3 to pre for tDCS (see Figure 2).

For AMT, multifactorial ANOVA revealed an effect of TIME [F(1.87) = 12.29, *p* ≤ 0.001, η^2^_p_ = 0.393], and an interaction of TIME and INTERVENTION [F(4.15) = 2.99, *p* = 0.022, η^2^_p_ = 0.136]. No effect for INTERVENTION was found [F(3) = 1.53, *p* = 0.217, η^2^_p_ = 0.074].

For RMT ANOVAs did not show any effects.

Taken together, tACS increased corticospinal excitability and the effect persisted for at least 1 h, while the effect of tRNS persisted at least for 15 min.

### Effects on conditioned motor evoked potentials

Raw data for TS (SICI, SAI, CBI) are given in Supplementary Table 1.

Comparing the absolute amplitudes of conditioned and unconditioned MEPs for SICI, SAI and CBI prior to the intervention a conditioning effect was present for SICI [F(1) = 91.125, *p* ≤ 0.001, η^2^_p_ = 0.827] and SAI [F(1) = 47.0314, *p* ≤ 0.001, η^2^_p_ = 0.712] but not for CBI [F(1) = 2.2164, p = 0.153, η^2^_p_ = 0.104].

Multifactorial ANOVA of the conditioned MEPs relative to the TS revealed, that there was no effect on SICI regarding the factors TIME [F(3) = 1.031, *p* = 0.386, η^2^_p_ = 0.051] or INTERVENTION [F(3) = 0.367, *p* = 0.777, η^2^_p_ = 0.019] nor interaction of factors [F(9) = 1.741, *p* = 0.671, η^2^_p_ = 0.038]. Also, for SAI there was no effect of TIME [F(3) = 2.560, *p* = 0.064, η^2^_p_ = 0.119] or INTERVENTION [F(3) = 0.742, *p* = 0.531, η^2^_p_ = 0.038] or interaction of factors [F(9) = 1.653, *p* = 0.104, η^2^_p_ = 0.080]. The same was the case for CBI. There was no effect of TIME [F(3) = 1.014, *p* = 0.393, η^2^_p_ = 0.051] or INTERVENTION [F(3) = 0.226, *p* = 0.878, η^2^_p_ = 0.012], and no interaction of factors [F(9) = 0.398, *p* = 0.935, η^2^_p_ = 0.021].

**FIGURE 3:**
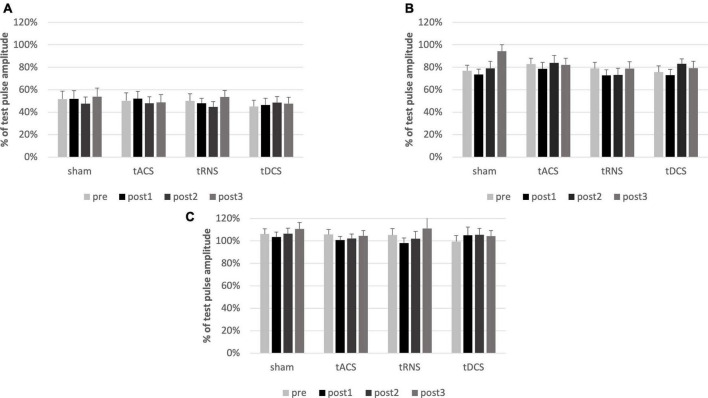
Analysis of SICI, SAI and CBI. Conditioned MEPs relative to the test pulses pre and post interventions are shown. Error bars representing the standard error of the mean. There is no effect of any cerebellar intervention on SICI **(A)**, SAI **(B)** or CBI **(C)**. SICI results in an inhibition of about 50%, SAI of about 20%. CBI does not influence the MEP. MEP, motor evoked potential; SAI, short-latency afferent inhibition; SICI, short-latency intracortical inhibition; CBI, cerebellar brain inhibition. post1 = 15 min; post2 = 55 min; post3 = 95 min post intervention.

To sum up, SICI, SAI, and CBI were not modulated by any type of intervention (see Figure 3A–C).

### Effects on movement trajectories

**FIGURE 4:**
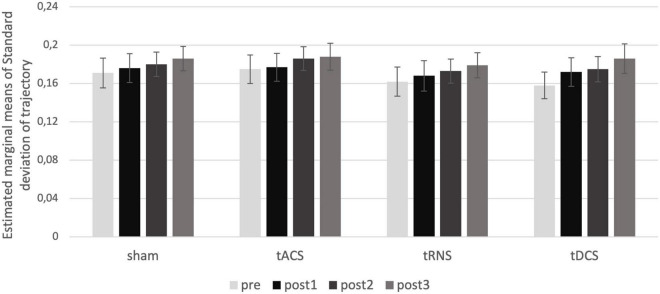
Intervention effect on movement trajectories. Estimated marginal means of standard deviation for the interaction of the factors TIME and INTERVENTION are shown. There is no effect of intervention over time. post1 = 15 min; post2 = 55 min; post3 = 95 min post intervention.

Regarding movement analysis, the standard deviation of movement trajectories was sensitive enough to represent expected differences in movement accuracy depending on velocity, handedness, and time (represented in significant effects regarding the factors TIME, HAND, VELOCITY, and DIRECTION; all significant results of the ANOVAs are presented in Supplementary Table 2, and estimated marginal means in Supplementary Image 1). However, there was no significant effect of the cerebellar stimulation INTERVENTION [F(3) = 0.4618, p = 0.710, η^2^_p_ = 0.026] (see Figure 4).

Regarding the bioinformatic modeling, in all experiments symmetric KL-divergences were between 0.5 and 2, which cannot be interpreted as a pronounced discrepancy, whereas a moderate level of difference should register the divergent value larger than 5. We also applied a sliding window approach on the time-normalized trajectories to examine the plasticity effect in a millisecond scale, i.e., a fractional part of the time-normalized trajectories. Also, in the sliding window approach, no significant effect of stimulation and no learning effect between the sessions was found in line with the observations in standard deviation.

The ANOVA regarding effects over time revealed a significant main effect for the factor DAY [F(1.9) = 12.073, *p* < 0.001, η^2^_p_ = 0.482] and the interaction of DAY and VELOCITY [F(1.81) = 9.487, *p* = 0.001, η^2^_p_ = 0.422]. This effect can be explained by an increasing inaccuracy over time in the fast movements condition, while the performance in the slow movement condition did not change (Supplementary Image 1). Since there is no improvement in performance, this finding speaks against a learning effect over time.

Taken together, there was no specific effect of any stimulation method on the movement trajectories.

### Side effect questionnaire

**FIGURE 5:**
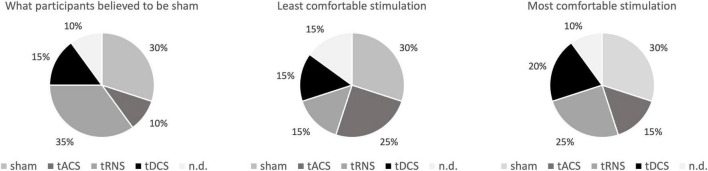
Post measurement questionnaire. Shown are the data of the subjects who were asked to rate which stimulation they thought do be sham and which they found most unpleasant or pleasant. In addition to the 4 interventions, the selection “I cannot decide” was possible. There was no difference between the methods regarding those parameters. tACS, transcranial alternating current stimulation; tDCS, transcranial direct current stimulation; tRNS, transcranial random noise stimulation; n.d., no decision, e.g., “I cannot decide”.

There were no relevant side effects. Only after one sham stimulation, a headache with an intensity of 4 on the visual analog scale was reported. Besides, one participant reported seeing flickering light when receiving tACS, which ended directly after the stimulation. When asked which day they believed they had received sham stimulation [X^2^(4) = 5.5; *p* = 0.24], as well as which method was the most [X^2^(4) = 4; *p* = 0.406] or least comfortable one [X^2^(4) = 2; *p* = 0.736], X^2^ goodness of fit test revealed an even distribution of answers between the methods. This suggests that no method was more or less comfortable than another, and that the participants could not identify sham stimulation as such (see Figure 5). Taken together, all tCS interventions appeared to be safe and well-tolerated.

## Discussion

To the best of our knowledge, this is the first sham-controlled study investigating different cerebellar tCS techniques intra-individually. The main finding of our study is that corticospinal excitability, reflected by the MEP amplitudes, is enhanced by tACS and tRNS. This effect lasted longer after cerebellar tACS stimulation i.e., for at least an hour. We found no effect of tDCS nor effects of any intervention on CBI, SICI, SAI, or the behavioral parameters.

Previous data on cerebellar tCS are heterogeneous. In line with our findings, an increase of corticospinal excitability after cerebellar tACS (Naro et al., 2016, 2017) but no change of CBI, SAI, or SICI was described previously (Doeltgen et al., 2016; Spampinato et al., 2021). Also, in keeping with our results, no effect of isolated anodal tDCS on motor cortex excitability has been found before (Behrangrad et al., 2019). However, some studies reported effects of tACS or tDCS on CBI, SICI, and behavioral parameters but used a study design that differed from ours regarding coil type (double cone coil), electrode size (larger), and stimulation parameters (Galea et al., 2009; Naro et al., 2017; Ates et al., 2018). For tRNS, no previous data on cerebellar application exists.

The mechanisms of how tACS and tRNS influence neural circuits are not fully understood. However, it has been hypothesized that tACS modulates neuronal firing rates through locking to their frequency and phase (Battleday et al., 2014). In a recent animal study, it has been shown that Purkinje cells could be entrained to external stimulation at certain frequencies (Asan et al., 2020). Probably, this represents a resonance effect as cells were stimulated with their natural/baseline frequency, e.g., 50 Hz. In humans, it has been shown that the stimulation effect of tACS is state-dependent, i.e., that changes of oscillatory activity were present when participants performed a task (Alagapan et al., 2016). Besides, effects of cerebellar 10 Hz tACS on the learning-related alpha power and premotor-cerebellar connectivity could be shown recently (Schubert et al., 2021). This underlines the relevance of network activity regarding oscillatory brain activity. However, network responses after tACS are variable and until now difficult to predict (Reato et al., 2010).

Two different hypotheses are postulated for tRNS effects with neuronal oscillations playing an important role. The first is based on the influences of natural oscillations by a phenomenon called “stochastic resonance” (van der Groen et al., 2019), i.e., that a non-linear system can be amplified by means of noise, which leads to periodic exceeding of the threshold (Gammaitoni et al., 1998). This in turn possibly leads to a higher sensitivity to weak external input (Moss et al., 2004). Alternatively, it was hypothesized that neurons with a sufficiently long time constant could be influenced by the summation of the stimulation effect of two or more following electric stimuli. This is particularly relevant when using hf-tRNS, which avoids the development of biochemical homeostasis during the stimulation due to an inconstant influence on the ion channels and consequently membrane potentials (Fertonani et al., 2011). Regarding neurochemical mechanisms, it has been shown that the effect of tRNS can be suppressed by benzodiazepines and depends on sodium channels, but not on NMDA receptors (Chaieb et al., 2015).

Probably, cerebellar tACS and tRNS reduce the inhibitory output of the cerebellum, which leads to an increase of excitability in cerebello-thalamo-cortical pathways. This was reflected by an increase of unconditioned MEP amplitudes in the present study. In animal studies, it has been shown that the cerebello-dentato-cortical pathway can have both inhibitory and excitatory effects on the motor cortex, mediated by different interneurons (Na et al., 1997; Holdefer et al., 2000). In addition to classical CBI that is thought to be caused by activation of Purkinje cells, which in turn inhibit the excitatory effect of cerebello-thalamo-cortical pathways and therefore lead to a decrease of corticospinal excitability, it has recently been hypothesized that a stronger activating effect of parallel fibers on Purkinje cells could also lead to an increase of CBI (Celnik, 2015; Spampinato et al., 2021). Taking this into account, there are different explanations for the excitability changes in our study. The stimulation could directly interact with the firing rate of the Purkinje cells. Even if one predicts a short-term increase in their activity due to a resonance effect after 50 Hz tACS, the long-lasting after effect could be a result of a *trans*-synaptic long-term depression-like effect, resulting from the modulation in network activity over 20 min and subsequent biochemical changes (D’Angelo, 2011). Alternatively, 50 Hz oscillations could interfere with the firing rates of granule cells. Granule cells are the only neurons with an excitatory output to the Purkinje cells and the molecular layer interneurons (D’Angelo, 2011). The stimulation could negatively influence their activating output over parallel fibers on the Purkinje cells, as their natural frequency lies in the theta band (5–7 Hz) (Gandolfi et al., 2013). This could also lead to an increase of corticospinal excitability due to the reduction of inhibitory influence of Purkinje cells on cerebello-thalamo-cortical pathways. The possibility of influencing Purkinje cells and/or inter-connected neuronal circuits non-invasively potentially has high clinical relevance, because especially the pathophysiology of alcohol-responsive movement disorders, e.g., essential tremor and myoclonus dystonia, seems to be related to activity of these cell types (Frucht and Riboldi, 2020; Madelein van der Stouwe et al., 2020). All these models explain an effect of stimulation of the cerebellum *via* direct or indirect cerebello-neocortical projections. In our study, we found an increase of MEP amplitudes but no effect on intracortical excitability in the motor cortex nor an effect on sensorimotor integration. The mechanism leading to the increase in MEP amplitudes may have occurred at the cortical level or in cerebellar spinal projections. This cannot be answered unequivocally based on the available data. As spinal excitability measures including direct current motor cortex stimulation, H-reflex and F-response were not measured in our study, the site of effective stimulation cannot be determined with certainty.

When interpreting our results, some limitations need to be considered. Given the lack of previous studies testing the effects of tRNS on the measures determined in the present study, the tRNS effects we found should be considered preliminary and have to be replicated in future studies. Regarding CBI, we aimed to compare cerebellar interventions in a representative sample. Therefore, it was important not to pre-select our participants. In a pilot study in our laboratory on CBI with a double-cone coil used for cerebellar conditioning and also in other published studies using this coil (Kassavetis et al., 2011), subjects did not tolerate the procedure due to pain and discomfort. Therefore, we used another published CBI protocol using a figure-of-eight coil, and no participant had to be excluded in our study. Although other studies (Carrillo et al., 2013; Brusa et al., 2014; Koch et al., 2014; Benussi et al., 2018, 2019) with similar experimental conditions found CBI, this was not the case in the present study. In general, it is still unclear if figure-of-eight coils are capable of activating Purkinje cells in the cerebellar cortex (Hardwick et al., 2014; Fernandez et al., 2018). Therefore, we cannot make any conclusions on the effect of cerebellar interventions on CBI.

Regarding SICI, in view of time constraints only one conditioning intensity was tested, so that recruitment curves could not be determined. Using lower intensities or recruitment curves might have revealed different effects.

We could show that the sensors detected variability changes of hand movements concerning speed, direction, handedness, and time course. However, there were no intervention-specific changes of behavioral performance. This is probably explained by the fact that the task we used was too simple and not specific enough to target cerebellar function. A sequence learning task might have been more sensitive. Including such a task, however, might have caused interference effects of (cerebellar) learning and plasticity induced by tCS. Even though we tried to avoid such effects, a possible influence of the motor task we used on our readouts cannot be excluded.

## Conclusion

Taken together, corticospinal excitability increased after cerebellar tRNS and tACS, but not following cerebellar tDCS. The effects of tACS lasted up to 1 h, i.e., longer than those of cerebellar tRNS. These findings are relevant for clinical applications of tCS.

## Data availability statement

The raw data supporting the conclusions of this article will be made available by the authors, without undue reservation.

## Ethics statement

The studies involving human participants were reviewed and approved by Ethikkommission der Universität zu Lübeck. The patients/participants provided their written informed consent to participate in this study.

## Author contributions

RH, AM, TB, and AW conceived the study design. TMB and RH acquired the data. TMB and HX preprocessed the data. RH and HX analyzed the data. RH, TB, MP, HX, ER, AM, TMB, and AW interpreted the data and revised the manuscript. RH drafted the manuscript. All authors approved the final version of the manuscript.

## Abbreviations

AMT: active motor threshold
ANOVA: analysis of variance
CBI: cerebellar brain inhibition
CS: conditioning stimulus
EMG: Electromyography
FDI: first dorsal interosseous muscles
IMU: inertial measurement units
ISI: interstimulus interval
KL-divergence: Kullback-Leibler divergence
M1: primary motor cortex
MEP: Motor evoked potential
NIBS: Non-invasive brain stimulation
ProMPs: Probabilistic Movement Primitives
RMT: resting motor threshold
tACS: transcranial alternating current stimulation
tCS: transcranial current stimulation
tDCS: transcranial direct current stimulation
(hf-)tRNS: (high-frequency) transcranial random noise stimulation
TMS: transcranial magnetic stimulation
TS: test stimulus
SAI: short-latency afferent inhibition
SICI: short-latency intracortical inhibition.
