# Supplementary material for: Cerebellar transcranial current stimulation – An intraindividual comparison of different techniques
Source: Front Neurosci. 2022 Sep 15;16:987472. doi: 10.3389/fnins.2022.987472 (PMC9521312; doi:10.3389/fnins.2022.987472)
Supplement: Supplementary file 1 [file Data_Sheet_1.DOCX]

**Post Measurement Questionnaire**

Subject code:

1-4. Measurement Day


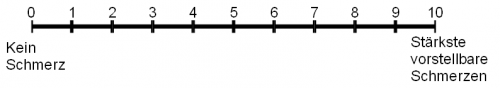


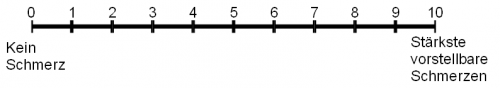
Did you experience headache during the measurement?


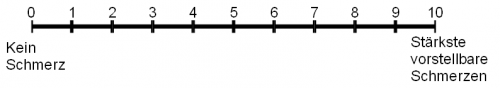
Did you experience headache after the measurement?


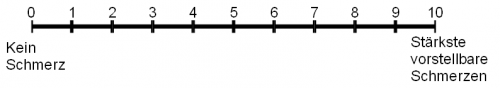
Did you experience malaise?


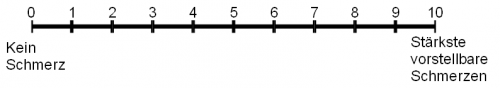
Did you experience vertigo?


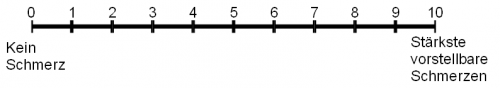
Did you feel sensations in your hand(s)?


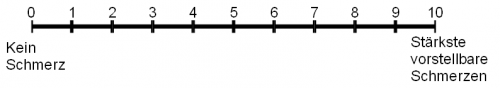
Did you feel a local heating?


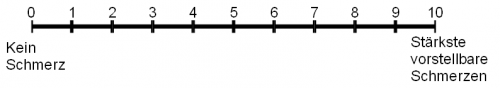
How tired were you before the measurement?


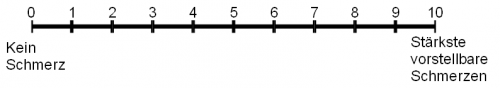
How tired are you after the measurement?

Did you experience difficulties during the movement
task after the stimulation?
(0 means ‘no difficulties’, 10 means ‘greatest difficulties imaginable’, if you found the task more simple, please refer to ‘other’)

Other:

On the last measurement day:

On which day did you receive the most comfortable stimulation?

On which day did you receive the least comfortable stimulation?

On which day do you think that you did not receive stimulation?
