# Supplementary material for: Cerebellar transcranial current stimulation – An intraindividual comparison of different techniques
Source: Front Neurosci. 2022 Sep 15;16:987472. doi: 10.3389/fnins.2022.987472 (PMC9521312; doi:10.3389/fnins.2022.987472)
Supplement: Supplementary file 2 [file Table_1.pdf]

Supplementary Table 1 TMS thresholds and test pulse intensities

	sham	*tACS*	*tRNS*	*tDCS*
**RMT, MSO (± SEM)**				
Pre	38 (± 1.81)	38 (± 1.71)	37 (± 1.55)	38 (± 1.56)
Post1	38 (± 1.81)	37 (± 1.54)	37 (± 1.49)	37 (± 1.56)
Post2	38 (± 1.75)	38 (± 1.67)	37 (± 1.47)	38 (± 1.57)
Post3	38 (± 1.73)	38 (± 1.52)	38 (± 1.56)	38 (± 1.55)
**AMT, MSO (± SEM)**				
Pre	31 (± 1.26)	31 (± 1.37)	30 (± 1.38)	30 (± 1.27)
Post1	31 (± 1.34)	29 (± 1.14)	30 (± 1.44)	31 (± 1.36)
Post2	32 (± 1.28)	30 (± 1.22)	31 (± 1.40)	31 (± 1.35)
Post3	32 (± 1.32)	31 (± 1.28)	31 (± 1.44)	31 (± 1.39)
**TS (MEP), MSO (± SEM)**	63 (± 3.81)	62 (± 3.56)	61 (± 3.53)	60 (± 3.32)
**TS (SICI, CBI, SAI), MSO (± SEM)**				
Pre	62 (± 3.94)	62 (± 3.57)	60 (± 3.49)	60 (± 3.55)
Post1	62 (± 3.81)	57 (± 3.97)	59 (± 3.45)	59 (± 3.63)
Post2	63 (± 3.77)	60 (± 3.37)	59 (± 3.38)	60 (± 3.55)
Post3	62 (± 3.81)	60 (± 3.42)	59 (± 3.44)	60 (± 3.42)

RMT = resting motor threshold; AMT = active motor threshold; TS = test stimulus; SICI = short-interval intracortical interaction; SAI: short-latency afferent inhibition; CBI: cerebellar brain inhibition; motor thresholds are expressed as the mean percentage of maximum stimulator output (MSO); SEM = standard error of mean
