# Supplementary material for: Cerebellar transcranial current stimulation – An intraindividual comparison of different techniques
Source: Front Neurosci. 2022 Sep 15;16:987472. doi: 10.3389/fnins.2022.987472 (PMC9521312; doi:10.3389/fnins.2022.987472)
Supplement: Supplementary file 3 [file Table_2.pdf]

ANOVA	Factor	p	F	η^2_p
Total (Factors: INTERVENTION, TIME, VELOCITY, DIRECTION, HAND)	Time	<.001	20.7509	0.550
	Velocity	<.001	93.4052	0.846
	Direction	<.001	189.0712	0.918
	Time * Velocity	<.001	15.5103	0.477
	Velocity * Direction	0.003	11.7077	0.408
Right Hand (Factors: INTERVENTION, TIME, VELOCITY, DIRECTION)	Time	<.001	15.985	0.485
	Velocity	<.001	82.813	0.830
	Direction	<.001	79.236	0.823
	Time * Velocity	<.001	11.509	0.404
Left Hand (Factors: INTERVENTION, TIME, VELOCITY, DIRECTION)	Time	<.001	17.702	0.496
	Velocity	<.001	101.910	0.850
	Direction	<.001	177.371	0.908
	Time * Velocity	<.001	13.363	0.426
	Velocity * Direction	0.002	13.790	0.434
Fast Movement (Factors: INTERVENTION, TIME, DIRECTION, HAND)	Time	<.001	18.448	0.520
	Direction	<.001	82.768	0.830
Slow Movement (Factors: INTERVENTION, TIME, DIRECTION, HAND)	Time	<.001	12.503	0.424
	Hand	<.001	91.339	0.843
	Direction	<.001	219.388	0.928

Supplementary Table 2: Detailed results of the ANOVAs of the standard deviation of movement trajectories. All (but only) significant results are presented.
