# Supplementary material for: Cerebellar transcranial current stimulation – An intraindividual comparison of different techniques
Source: Front Neurosci. 2022 Sep 15;16:987472. doi: 10.3389/fnins.2022.987472 (PMC9521312; doi:10.3389/fnins.2022.987472)
Supplement: Supplementary file 4 [file Image_1.pdf]

Supplementary Figure 1

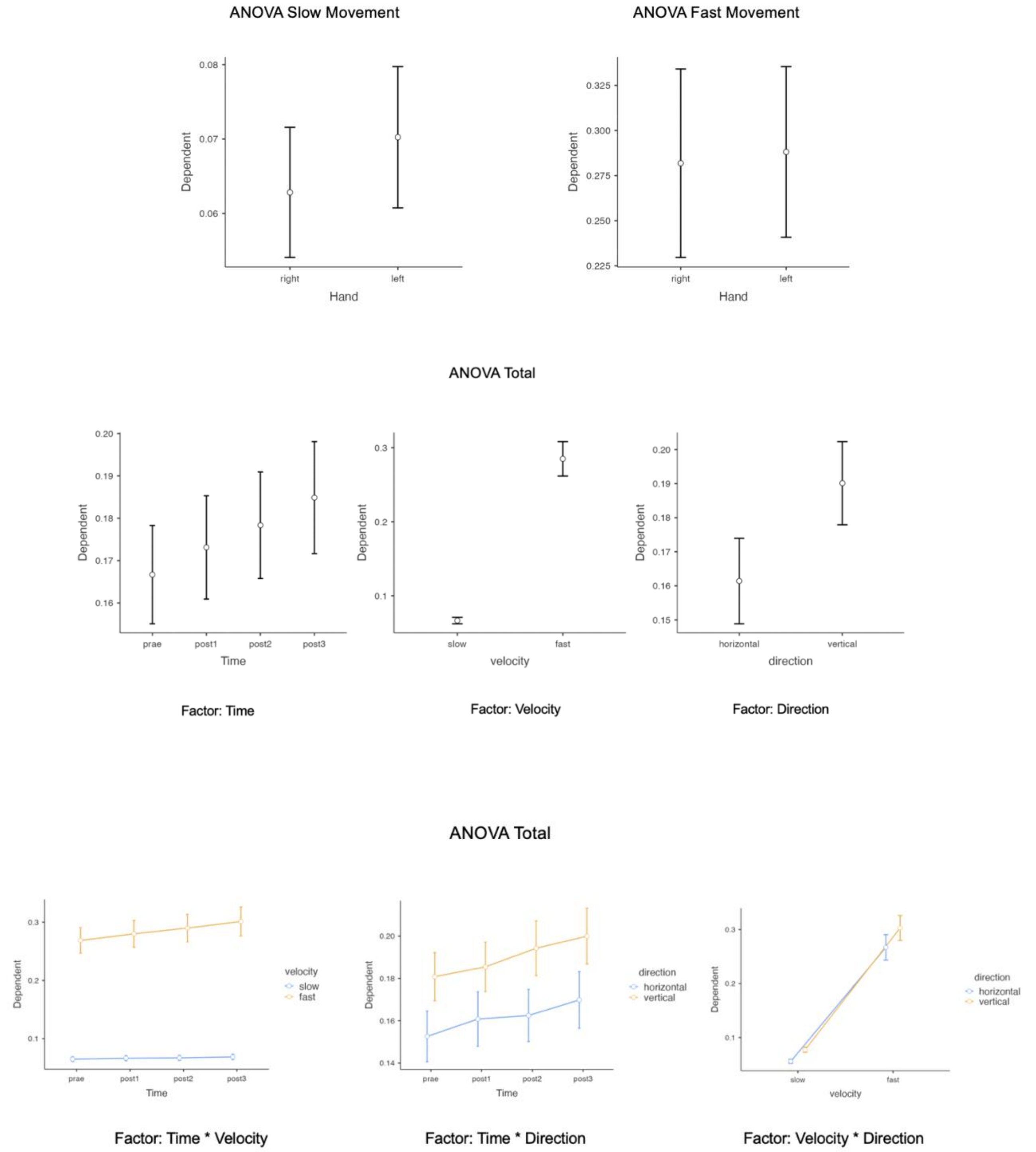

Estimated marginal means of standard deviation. For slow movements, the difference between right and left hand is significant whereas in fast movements no significant difference could be found. In the total ANOVA, significant increase of standard deviation can be observed after time, as well as fast movements show a higher standard deviation than slow movements and movements in vertical direction result in a higher standard deviation than horizontal movements. For combined effects can be seen that the main effects Time x Velocity and Velocity x Direction are strongly influenced by the effect of movement velocity, while the main effect Time x Direction seems to be a combined effect.
