# Supplementary material for: Cerebellar transcranial current stimulation – An intraindividual comparison of different techniques
Source: Front Neurosci. 2022 Sep 15;16:987472. doi: 10.3389/fnins.2022.987472 (PMC9521312; doi:10.3389/fnins.2022.987472)
Supplement: Supplementary file 5 [file Image_2.pdf]

Supplementary Figure 2

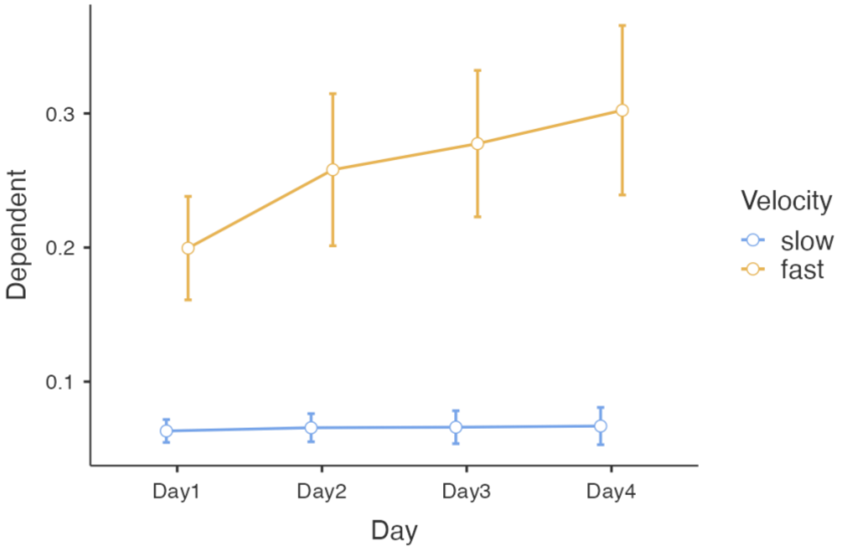

The effects of the ANOVA on the pre-interventional data (standard deviation of movement trajectories) for the slow and fast conditions are displayed.
